# Supplementary material for: Sprint performance and force application of tennis players during manual wheelchair propulsion with and without holding a tennis racket
Source: PLoS One. 2022 Feb 4;17(2):e0263392. doi: 10.1371/journal.pone.0263392 (PMC8815940; doi:10.1371/journal.pone.0263392)
Supplement: Attachment — Submitted filename: Response to Reviewers.pdf [file pone.0263392.s001.pdf]

20 October 2021

Ilona ALBERCA
IAPS research unit
Faculty of STAPS
South University of Toulon, Building Z1
La Garde Campus
CS 60584
83041 TOULON CEDEX 9

Email: ilona.alberca@univ-tln.fr

Dear reviewers,

Thank you for your work and your feedback on this article *"Sprint performance and force application of tennis players during manual wheelchair propulsion with and without holding a tennis racket"*. Here are the answers I was able to give to each of your comments.

Response reviewer 2:

Thank you for your comments, which help to improve the article, particularly on its methodology. Regarding the main concern of the study, we have a small population. I added description of this limitation in the discussion. In addition, a statistical power test based on the reference article by de Groot et al. (2017) was carried out and determined that 8 subjects are sufficient in our study and in the statistical tests we carried out. This point was enhanced in discussion and methodology.

Based on your comments, I also incorporated several elements into the summary, including participant criteria, a reference for the study design, and a message of recommendations.

I clarified the meaning of the study in the introduction as you advised me.

Finally, concerning the methodology and the SPICES criteria, our article is an exploratory research article. Consequently, the methodology used during the experiments cannot fully correspond to the SPICES components. In order to incorporate this remark, I have nevertheless modified this part and added elements to best respond to your comment. I hope this will suit you.

Response reviewer 3:

Thank you for your very constructive and motivating feedback.

Regarding the statistical part, it has been corrected according to your advice. After consulting several experts, my choice fell on a multivariate test to ensure that there was a significant difference between the parameters measured depending on the test condition. Once this test was validated, I refined the variable-to-variable analysis with a T-test. I think the combination of these two tests strengthens our statistics and best matches the comparison we want to make based on the data we have. I added the information in the statistical analysis. In addition, due to the exploratory nature of this research and the statistics, I modulated the results to be conditional and not affirmative.

Finally, I added some clarifications. Hence the participants used their own manual tennis wheelchairs. I only have the measurement of the size of the wheelchair wheels of each athlete, but without having everyone's camber angle, we know that athletes all had a minimum angle of 18 °. I can add this clarification. Otherwise, I wrote a section in limitations regarding the lack of knowledge of chair measurements. While this is a limitation, it is also a strength to our study. Indeed, we evaluated the athletes into their own wheelchairs with the same settings as those used in competition. We are therefore as close as possible to an experiment in ecological condition.

I hope you find these answers and revisions satisfactory. I remain at your disposal to answer any questions you may have.

Your sincerely,

Ilona Alberca
